# Sequential Proximal Tibial Stress Fractures associated with Prolonged usage of Methotrexate and Corticosteroids: A Case Report

**DOI:** 10.5704/MOJ.1511.010

**Published:** 2015-11

**Authors:** TJL Tan, WLS Ho

**Affiliations:** Department of Orthopaedic Surgery, Tan Tock Seng Hospital, Singapore

**Keywords:** Tibial stress fracture, total knee arthroplasty, methotrexate osteopathy

## Abstract

Stress fractures of the proximal tibia metaphysis are rare in the elderly. We present a case of a 65-year old male who developed sequential proximal tibia stress fractures associated with prolonged usage of methotrexate and prednisolone within a span of 18 months. Magnetic Resonance Imaging revealed an incomplete stress fracture involving the medial proximal tibial region. The patient was treated with stemmed total knee arthroplasty (TKA) bilaterally. Stress fractures should be considered in patients with atypical knee pain who have a history of methotrexate and prednisolone usage. TKA is an effective treatment in stress fractures of the proximal tibia.

## Introduction

Stress fractures can be caused by either abnormal repetitive stresses placed on normal bone, or by normal stresses placed on abnormal bone^1^. Causes of abnormal bone include osteoporosis, inflammatory conditions such as rheumatoid arthritis as well as prolonged usage of medications such as corticosteroids and methotrexate. Prolonged methotrexate usage has been postulated to induce bone changes and predispose patients to stress fractures^2–4^. Whilst proximal tibial stress fractures have been associated with moderate to severe osteoarthritis of the knee, the association with mild osteoarthritis remains rare^5^. We describe a rare case of sequential proximal tibia stress fractures in a male patient on long-term methotrexate and prednisolone with mild osteoarthritis of the knees. The authors have obtained the patient’s informed written consent for print and electronic publication of the case report.

## Case Report

**Fig. 1:**
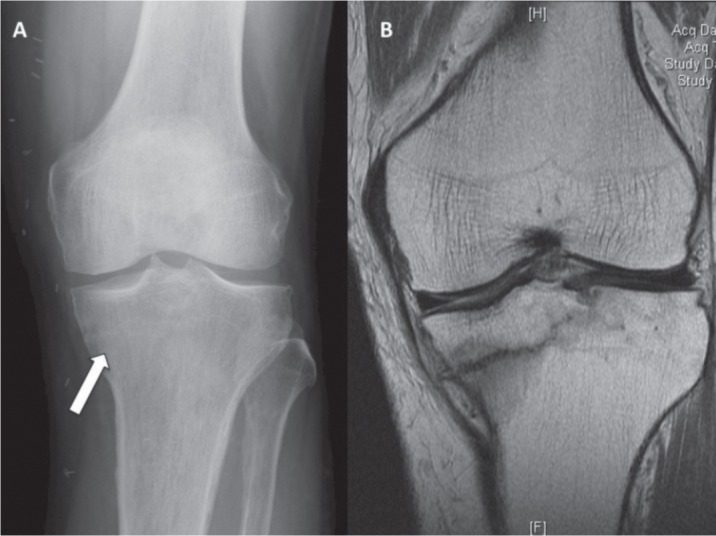
(A) Weight bearing X-ray of the left knee showing lucency over medial proximal tibia. (B) MRI of the left knee revealing a fracture of the medial aspect of the tibial plateau.

A 65 year-old man presented with a 3-month history of left knee pain after hiking. The patient had a known history of psoriasis for which he was treated with 15mg/week of oral methotrexate for the last four years. He also had a history of gout for which he was on 4mg of oral prednisolone daily, with colchicine for acute exacerbations. The patient developed a mechanical left knee pain after an episode of prolonged walking. There was no history of trauma. The patient was able to ambulate without aid, with complete resolution of pain on rest. Clinical examination revealed a left knee range of motion of 0 to 130º, with no effusion, palpable tenderness or warmth. Radiographs revealed a lucent line in the medial tibial plateau, suspicious of a stress fracture. Magnetic Resonance Imaging (MRI) (Figure 1.), confirmed the diagnosis of an incomplete proximal tibial stress fracture.

**Fig. 2:**
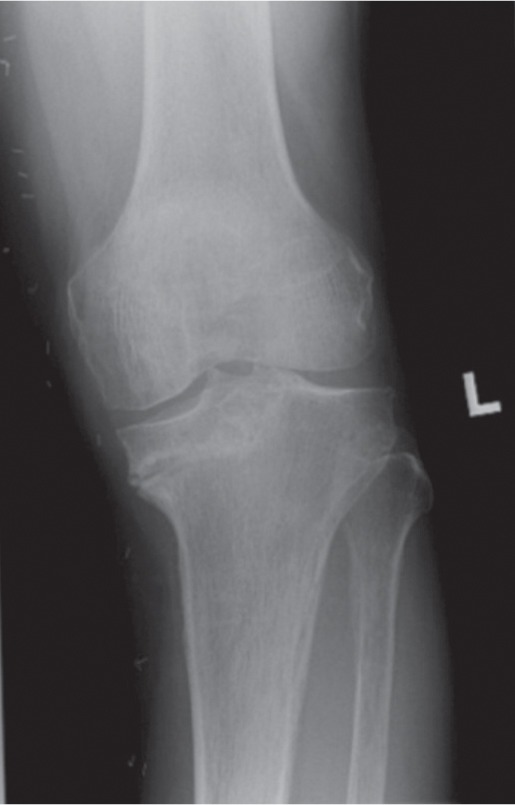
Weight bearing radiograph of the left knee showing progressive varus collapse of the medial tibial plateau.

The patient was treated non-surgically with a knee ranger brace and advised to reduce loading on the affected limb. However, the patient returned seven months later complaining of persistent pain. Repeat radiograph (Figure 2.) showed progressive collapse of the medial tibial plateau. In view of the progressive deformity and pain, the patient underwent a computer-navigation assisted left TKA. A stemmed tibia implant was used to compensate for the loss of medial support. Post-operatively, the patient achieved a good range of movement from 0-120º. Knee Society Scores and Knee Society Function Scores improved from 59 to 94, and 60 to 100 respectively at three months.

**Fig. 3:**
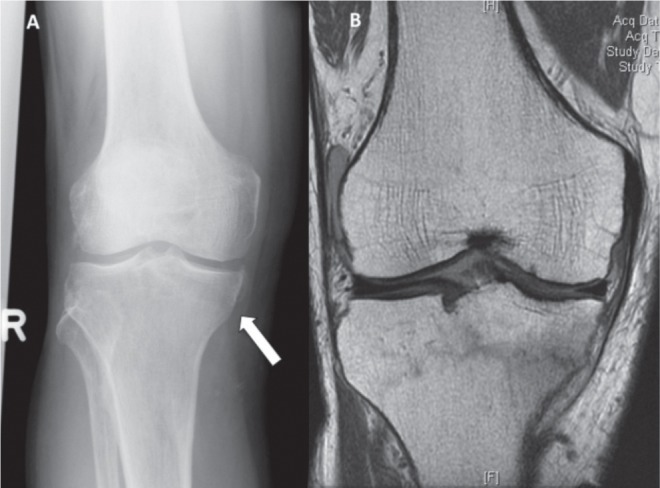
(A) Weight bearing X-ray of the right knee showing lucency over medial proximal tibia. (B) MRI of the right knee revealing a fracture of the medial aspect of the tibial plateau.

**Fig. 4:**
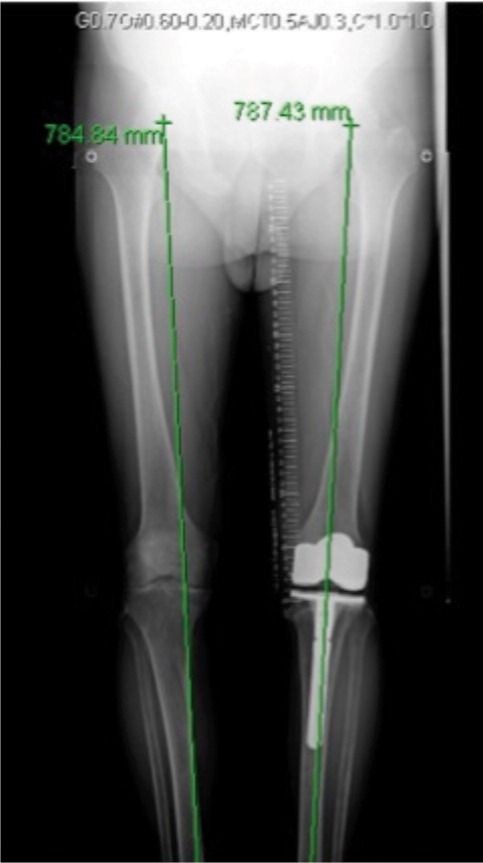
Long lower extremity radiograph showing minimal mal-alignment of the right knee with left total knee arthroplasty.

The patient returned 18 months later complaining of similar pain in the right knee. There was no history of trauma. Clinical examination revealed right knee range of motion of 0 to 130º, with no palpable tenderness. Radiographs and subsequent MRI revealed a right proximal tibial stress fracture. (Figure 3). Lower limb extremity radiographs did not demonstrate any significant varus/valgus malalignment. (Figure 4A-B.) A bone mineral densitometry utilizing dual energy x-ray absorptiometry (DEXA) revealed osteoporosis, with a Z score of -3.8 (Lumbar) and -3.2 (Proximal femur). At that point, he was not on any osteoporosis treatment. Calcium and vitamin D levels were normal.

**Fig. 5:**
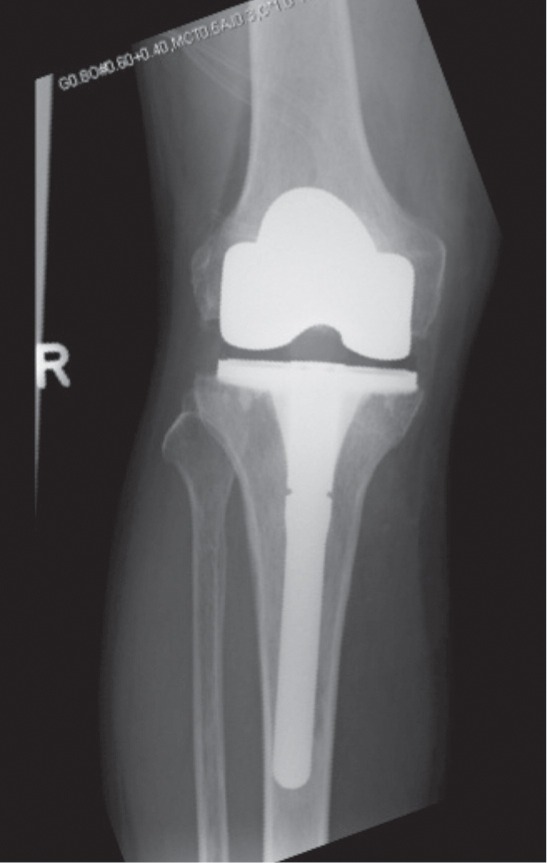
Post-operative antero-posterior (AP) X-ray of the right knee showing a total knee arthroplasty with stemmed tibial implant.

Computer-assisted navigation TKA of the right knee was performed. (Figure 5). Post-operatively, the patient achieved a good range of movement from 0-120º. Knee Society Scores and Knee Society Function Scores improved from 54 to 80, and 30 to 100 respectively at 3 months. The patient was started on strontium ranelate for osteoporosis. The patient was reviewed two years after surgery and remained well.

## Discussion

Proximal tibia stress fractures may occur on the background of osteoarthritis, due to the inherent mal alignment of the knee joint. However, it is rare for stress fractures of the proximal tibia to occur in cases of mild osteoarthritis of the knee, where there is minimal mal alignment. In our case, we believe that the stress fractures occurred in association with methotrexate osteopathy due to prolonged usage of methotrexate (MTX). MTX is an anti-metabolite that inhibits RNA and DNA synthesis^3^. MTX is postulated to affect osteoblastic activity and may enhance osteoclastic bone resorption. Prolonged usage of MTX can result in osteopathy^4^. In addition, our patient was on long-term prednisolone, which may have contributed to osteoporosis. MTX and prednisolone usage may account for a weakened bone in our patient, predisposing the proximal tibia to stress fracture on the background of a mild osteoarthritis.

Patients with stress fractures of the proximal tibia may present without any preceding event of trauma. Often, they present with sudden onset of pain aggravated by activity and relieved by rest^6^. On physical examination, they may be an effusion and pain on palpation. Plain radiographs can reveal abnormalities such as a sclerotic band or lucency but these changes can be subtle, especially in acute stress fractures. Osteophyte formation may obscure bony changes^6^. Magnetic Resonance Imaging (MRI) can be used to confirm the diagnosis and has the added advantage of being able to evaluate both the osseous structures and soft tissues in the knee. This may be important when considering arthroplasty as a form of treatment.

Treatment options for proximal tibia stress fractures can be surgical or non-surgical. Non-surgical modalities include a period of offloading, complete non-weight bearing or partial weight bearing. This may be complemented by immobilization in either a cast or a brace to protect the fracture site. Stress fractures can often heal after a period of reduced activity. However, complications of such treatment include non-union, mal-union or varus collapse of the tibial plateau, which can worsen the mal-alignment, leading to increased pain. Surgery is indicated if patients present with worsening pain and deformity. Arthroplasty can treat the underlying arthritis, correct deformity and provide immediate stability. Mal alignment can be corrected and abnormal stresses on the tibia can be relieved. In our patient, we opted for computer navigation to improve accuracy of bone cuts and correction of deformity. A stemmed tibial implant can reduce stresses and provide additional stability.

## Conclusion

Prolonged usage of methotrexate and corticosteroids can predispose patients to stress fractures. In such patients who present with persistent knee pain, the diagnosis of stress fracture should be considered. Magnetic resonance imaging can be utilized to confirm diagnosis. Total knee arthroplasty is an effective treatment option that reduces pain, corrects deformity and allows for immediate post-operative ambulation.
